# Function of reactive oxygen species in myeloid-derived suppressor cells

**DOI:** 10.3389/fimmu.2023.1226443

**Published:** 2023-08-14

**Authors:** Jiaojiao Huang, Yue Zhao, Kexin Zhao, Kai Yin, Shengjun Wang

**Affiliations:** ^1^Department of Laboratory Medicine, The Affiliated People’s Hospital, Jiangsu University, Zhenjiang, China; ^2^Department of General Surgery, Affiliated Hospital of Jiangsu University, Zhenjiang, Jiangsu, China; ^3^Department of Immunology, Jiangsu Key Laboratory of Laboratory Medicine, School of Medicine, Jiangsu University, Zhenjiang, China

**Keywords:** MDSCs, ROS, immunotherapy, tumor, tumor micro environment (TME)

## Abstract

Myeloid-derived suppressor cells (MDSCs) are a heterogeneous myeloid cell population and serve as a vital contributor to the tumor microenvironment. Reactive oxygen species (ROS) are byproducts of aerobic respiration and are involved in regulating normal biological activities and disease progression. MDSCs can produce ROS to fulfill their immunosuppressive activity and eliminate excessive ROS to survive comfily through the redox system. This review focuses on how MDSCs survive and function in high levels of ROS and summarizes immunotherapy targeting ROS in MDSCs. The distinctive role of ROS in MDSCs will inspire us to widely apply the blocked oxidative stress strategy in targeting MDSC therapy to future clinical therapeutics.

## 1 Introduction

Myeloid-derived suppressor cells (MDSCs) are a heterogeneous population of myeloid cells with immunosuppressive activity. MDSCs play a crucial role in tumorigenesis and inhibit antitumor immune responses to promote tumor development (1, 2). In addition to cancer, MDSCs are also involved in autoimmune diseases, sepsis, bone marrow transplantation and infection diseases (1, 3).Reactive oxygen species (ROS) have miscellaneous effects and are involved in both cell biological activities and oxidative stress disease (4). Notably, ROS are one of the dominant immunosuppressive functional effector molecules of MDSCs, and MDSCs can also adjust the ROS level to a proper level to maintain the state of MDSCs. Currently, immunotherapy that targets MDSCs has achieved significant results, but targeting ROS in MDSCs has not yet become a therapeutic focus that will be worth further investigation.

This paper summarizes the distinctive regulation, scavenging and effects of ROS in MDSCs. In addition, we generalize immunotherapy that targets ROS in MDSCs. This will provide novel potential insight for targeting MDSC immunotherapy.

## 2 MDSCs

Myeloid-derived suppressor cells (MDSCs) are a heterogeneous population composed of immature myeloid cells (IMCs). In pathological conditions such as cancer, infectious diseases, trauma, and some autoimmune disorders, IMCs cannot differentiate into mature myeloid cells, which causes the activation and expansion of MDSCs (1, 5). At present, we can identify MDSCs by phenotype and immunosuppressive function. The phenotype of mouse MDSCs is CD11b^+^Gr-1^+^. According to different epitopes (Ly6G and Ly6C) in Gr-1, mouse MDSCs can be further divided into two subgroups: CD11b^+^Gr-1^+^Ly6G^high^Ly6C^low^granulocyte/polymorphonuclear MDSCs (G-MDSCs/PMN-MDSCs) and CD11b^+^Gr-1^+^Ly6G^low^Ly6C^high^monocytic MDSCs (M-MDSCs) (6). More importantly, we can use different antiapoptotic molecules to discriminate PMN-MDSCs and M-MDSCs. The antiapoptotic molecule MCL-1 is required for the development of PMN-MDSCs, while M-MDSCs require another antiapoptotic molecule, c-FLIP (7). The phenotype of human MDSCs and their subsets is different from that of mice. Human MDSCs express CD11b^+^CD33^+^HLA-DR^-/low^. Human PMN-MDSCs express CD15, while human M-MDSCs express CD14 (8). In most types of cancer, PMN-MDSCs are the predominant population, while M-MDSCs have stronger immunosuppressive activity than PMN-MDSCs (1). Except for phenotypic identification, many original methods are being exploited to identify MDSCs. Single-cell RNA sequencing (scRNAseq) technology could describe MDSCs by novel surface markers (CD84, JAML) and definite PMN-MDSCs with enrichment genes (Ngp, Ltf, Anxa1, Mmp8 and Cybb) (9, 10). IHC staining analysis showed that MDSCs are located in the tumor epithelial border (11). Moreover, metabolite and lipid analyses of MDSCs also demonstrated that MDSCs have a specific response to high glucose concentrations (12).

The immunosuppressive activity of MDSCs relies on the expansion and activation of MDSCs. There are a variety of factors accounting for the expansion of the MDSCs, such as cyclooxygenase 2 (COX2), prostaglandin, stem-cell factor (SCF), macrophage CSF (M-CSF), granulocyte/macrophage CSF (GM-CSF), vascular endothelial growth factor (VEGF), TNF-α, polyunsaturated fatty acids, MyD88 and HIF-1α (13–15). Most of these factors advance the expansion of the MDSCs by triggering the STAT3, IRF8, C/EBP-β and NOTCH signaling (16). Among them, STAT3 is a vital regulator of the expansion of MDSCs, which can also upregulate the proinflammatory protein S100A8/9 expression and induce the expression of the downstream targets of STAT3 including survivin, BCL-XL and cyclin-D1 (13, 14). Endoplasmic reticulum (ER) stress can promote the accumulation of MDSCs by activating TNF-related ligand receptors which induce the apoptosis (15). Last but not least, the metabolites adenosine, IDO and lactic acid accumulated in the TME can also contribute to the expansion of MDSCs (17). Other factors, such as IFN-γ, IL-1β, IL-4, IL-6, IL-13, TNF and high mobility group Box 1(HMGB1), could influence the MDSCs suppressive activity by activating STAT1, STAT3, STAT6 and NF-κB signaling pathways (18).

Several major molecules contribute to MDSC-mediated immune suppression, including arginase 1 (Arg-1), inducible nitric oxide synthase (iNOS), COX2, TGF-β, IL-10 and ROS. Many factors, such as STAT3, C/EBPβ, p50 NF-κB, and IDO1, play a critical role in MDSC function by regulating these functional effector molecules (16, 19, 20). Arg-1, which converts L-arginine to urea and L-ornithine, inhibits T-cell function by decreasing the expression of the CD3ζ chain and impairing the expression of cyclin D3 and cyclin-dependent kinase 4 (cdk4) (21). ROS are the characteristic molecules of PMN-MDSCs, while M-MDSCs mainly produce NO (8). NO produced by MDSCs leads to the suppression of T-cell responses by reducing the tyrosine phosphorylation of JAK3 and STAT5, preventing MHC II transcription and triggering T-cell apoptosis (22). The interaction between ROS and NO can promote the formation of peroxynitrite, which leads to the desensitization of T-cell receptors and T-cell tolerance. Treatment of cancer with AT38 ([3-(aminocarbonyl)furoxan-4-yl] methyl salicylate) could increase antitumor immunity by interfering with the expression of ARG1 and NOS2 enzymes in myeloid cells (23).

In addition, MDSCs can recruit and expand Treg cells *via* the immunosuppressive cytokines IL-10 and TGF-β. MDSCs can also reduce the secretion of IL-6 and TNF-α by macrophages and shape them into the M2-type phenotype, which promotes tumor progression (24). In turn, Treg cells induce the expression of B7 homolog 1 (B7-H1), B7-H3 and B7-H4 on the cell surface of MDSCs, which causes an increase in IL-10 production and immunosuppressive activity of MDSCs (25). In addition, MDSCs can produce adenosine due to the high expression of CD73 and CD39, which hydrolyze ATP into adenosine, and adenosine can inhibit the immune responses of both T cells and NK cells in the tumor microenvironment (26).

## 3 ROS

Reactive oxygen species (ROS), oxygen-containing derivatives, include a range of species such as superoxide (O^2.-^), hydrogen peroxide (H_2_O_2_), nitric oxide, peroxynitrite, hypochlorous acid, singlet oxygen and hydroxyl radicals (27). Among them, the three most common forms of ROS are superoxide, H_2_O_2_ and hydroxyl. Different forms of ROS can have different targets. To illustrate, H_2_O_2_ takes effect through the modification of specific cysteine, selenocysteine, methionine and histidine residues in targeted proteins (28, 29), but O^2.-^, hydroxyl radicals and peroxynitrite can irreversibly undermine intracellular proteins, DNA and lipids (30). In cancer, the most studied ROS components are O^2.-^and H_2_O_2_ (31). However, the main increased pool of ROS released by MDSCs is primarily H_2_O_2_ under pathological conditions (32).

ROS are byproducts of aerobic respiration that can be produced by many cells, including hematopoietic stem cells (HSCs), tumor cells, cancer stem cells (CSCs) and immune cells (33). The production of ROS relies on cell type. Tumor cells, MDSCs and professional phagocytes can produce abundant ROS. However, HSCs and CSCs have low ROS content (34, 35).

ROS are short-lived, strong-effect and short reaction distance compounds that serve as a double-edged sword that elicits both beneficial and harmful effects in cells. The most common influence is the toxic side effects of ROS. Elevated levels of ROS can damage cells and intracellular components, cause DNA hydroxylation, protein denaturation and tissue damage, and ultimately lead to cell cycle G2/M arrest, apoptosis, senescence and death, and ROS can also participate in mitochondria, death receptors, and endoplasmic reticulum-mediated apoptosis (36). However, ROS also serve as the second messenger of cell signal transmission to play a regulatory role in many crucial biological activities of normal cells (4).

## 4 Sources of ROS in MDSCs

NADPH oxidase (NOX) enzymes and mitochondria are major sources of endogenous ROS. In addition, there are numerous cellular sources of ROS, including xanthine oxidase, cyclooxygenases, cytochrome p450 enzymes, lipoxygenases and the endoplasmic reticulum (28).

Two major sources of ROS in MDSCs are NOX2 and mitochondria. Compared with MDSCs, cancer cells and macrophages also utilize mitochondria and NADPH oxidase to produce ROS. However, T cells express no or very low levels of NADPH oxidase (37).

Mitochondria have ten sites to generate O^2.-^, particularly those derived from mitochondrial electron transport chain (ETC) complexes. Complex I and III of the ETC generate O^2.-,^ which is rapidly converted to H_2_O_2_ *via* mitochondrial SOD2, while the O^2.-^ from the complex can be converted into H_2_O_2_ by cytosolic SOD1 (38). Mitochondrial ROS are implicated in diverse diseases, including cancer, diabetes and inflammatory disorders, and regulate healthy cell physiological function (39).

The NOX family has seven members: NOX1–5, DUOX1 and DUOX2 (40). NOX2 is a multicomponent complex that is made up of a transmembrane heterodimer that contains NOX2 and p22phox. Other components are cytosolic protein factors, including p47phox, p67phox, p40phox and small GTP-binding proteins such as G proteins RAC1 or RAC2. Under basal conditions, gp91phox and p22phox are transmembrane proteins, while the cytosolic subunits p47phox, p67phox and p40phox are connected together, and RAC combined with GDP forms a complex with its inhibitor Rho-GDI and does not interact with the other three cytosolic subunits. When exposed to stimulus, NOX2 is activated. Upon activation, p47phox is phosphorylated and then migrates to the membrane, where it combines with gp91phox and p22phox. Rho-GDI separates from the complex, and then RAC binding with GDP combines with gp91phox to form a multicomponent complex (41). NOX2 catalyzes the conversion of oxygen molecules into superoxide anions, which generates H_2_O_2_ by SOD. Deficiency or dysfunction of NOX2 in phagocytes may reduce ROS production, resulting in chronic granulomatous disease (CGD) (42). Comparably, MDSCs in NOX2-deficient mice produced less ROS, which lose the ability to inhibit the CD8^+^ T-cell immune response (43). Rats and mice with decreased ROS caused by allelic polymorphisms of p47phox were more susceptible to developing severe arthritis (44).

## 5 Regulation of ROS production in MDSCs

Many factors can regulate ROS production, such as GM-CSF, interleukin, TGF, TNF, FGF, platelet-derived growth factor, TLR agonists, protease, nucleotide receptors, TCR stimulation and peroxynitrite (32, 45, 46).

Various types of cells and survival environments possess different ROS regulatory mechanisms. In terms of MDSCs, it has been proven that multiple molecules can govern intracellular ROS, such as STAT3, fatty acid transport protein 2 (FATP2) and noncoding RNAs. STAT3 is an important transcription factor related to the expansion, differentiation and function of MDSCs. STAT3 directly increases the expression of p47phox, which belongs to the NOX2 complex, by binding to the promoter region of p47phox. Blocking STAT3 could downregulate the expression of gp91phox and p47phox to decrease ROS production (43, 47, 48). In addition, tumor-derived GM-CSF activated STAT3 signaling to induce the expression of FATP2 in MDSCs. Subsequently, FATP2 in MDSCs can take up abundant lipids that cause elevated ROS levels (49). Furthermore, noncoding RNAs (lincRNAs and miRNAs) that have been upregulated during bacterial and viral infection are reported to influence ROS generation in MDSCs (50). During virus infection, lncRNA RUNXOR and HOTAIRM1 are upregulated and are responsible for elevated levels of ROS, Arg-1 and iNOS in MDSCs (51, 52). MiRNA-10a and miRNA-21, which are also upregulated in hypoxia-induced glioma-derived exosomes, could strengthen ROS and NO production in MDSCs with the potential to enhance the suppressive activity of MDSCs (53).

In addition, cancer-associated fibroblasts (CAFs) can polarize monocytes to MDSCs, which suppress CD8^+^ T-cell proliferation and function by generating ROS (54). Murine olfactory ecto-mesenchymal stem cell-derived exosomes could also enhance the suppressive activity of MDSCs by upregulating ROS and NO levels (55).

In contrast to MDSCs, other myeloid cells, such as macrophages, can stimulate NADPH oxidase expression and activity to elevate the level of ROS by other disparate factors, such as P2X7, brain-specific angiogenesis inhibitor 1 (BAI1), beryllium, myocardin-related transcription Factor A (MRTF-A) and TLRs (56–60). However, mitochondrial uncoupling protein 2 (UCP2), paraoxonase 1 (PON1) and IL-10 negatively regulate the ROS level in macrophages (61–63).

## 6 ROS scavenging in MDSCs

In general, the cell needs an appropriate level of ROS to maintain normal physiological function. Either too few or too many ROS are harmful. Normally, ROS production is controlled in a safe range, and superfluous ROS can be neutralized by the antioxidant system to maintain cell homeostasis. The antioxidant system contains antioxidant enzymes and nonenzymatic molecules. Common antioxidant enzymes include superoxide dismutase (SODs), catalase, peroxidase (PRDXs), peroxiredoxins (Prxs) and glutathione peroxidase (GPXs) (64). Other nonenzymatic antioxidant molecules are glutathione, flavonoids, thioredoxin, and vitamins A, C and E (65, 66). If the redox system is out of balance, the rising ROS will lead to oxidative stress. Oxidative stress is considered a vital inducer of many pathological diseases, such as cancer, atherosclerosis, multiple sclerosis, ischemia and reperfusion injury, Alzheimer’s disease, cardiovascular diseases and traumatic brain injury (67–71).

For example, the antioxidant system of tumor cells can cope with the production of ROS properly *via* antioxidant enzymes and autophagy (72). The overproduction of ROS in tumor cells could maintain the pro-tumourigenic signaling, which results from the upregulation of SOD expression, local inactivation of a H_2_O_2_-degrading enzyme, oxidative inactivation of phosphatase and tension homolog (PTEN) and mutations in Nrf2 and P53 transcription factors (4, 73–75).

**Figure 1:**
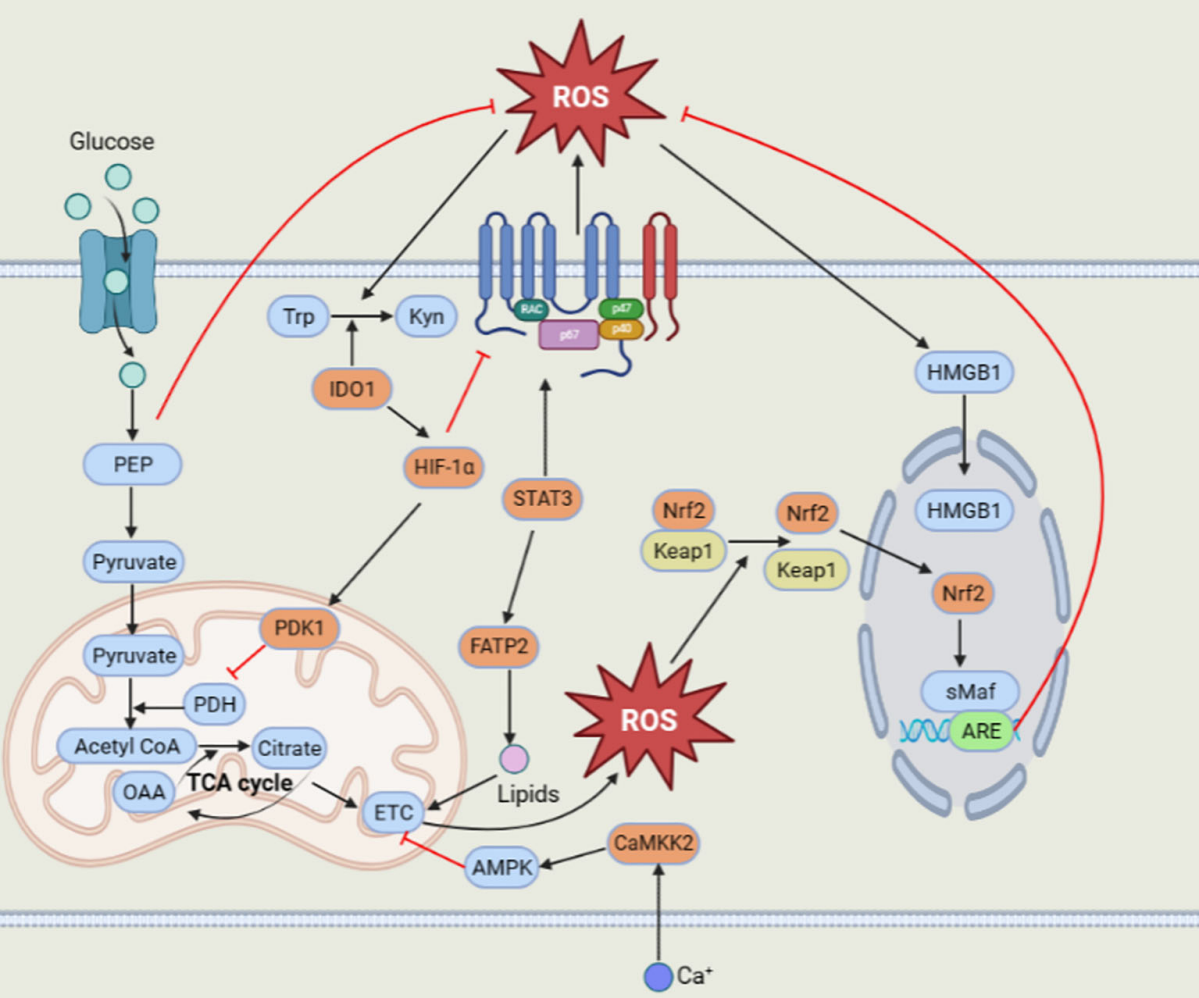
Regulation of ROS in MDSCs.

Surprisingly, MDSCs could still survive and function excellently by producing high levels of ROS. How can MDSCs scavenge superfluous ROS? This can be ascribed to some essential factors, such as Nrf2, HMGB1, IDO1, calcium-calmodulin kinase 2 (CaMKK2), HIF-1α, pyruvate dehydrogenase kinase 1 (PDK1) and phosphoenolpyruvate (PEP) (**Figure 1**)

The Nrf2 transcription factor plays a crucial role in regulating the antioxidative response and inducing the expression of antioxidant and detoxification enzyme genes, including heme oxygenase-1 (HO-1), NAD(P)H:quinone oxidoreductase 1 (NQO1), catalase and SOD (76). Under normal circumstances, Nrf2 combined with Kelch-like ECH-associated protein 1 (Keap1) is limited to degradation in the cytoplasm. However, under oxidative stress conditions, Keap1 is modified at a specific cysteine position to disable its E3 ligase adaptor and release Nrf2. The released Nrf2 translocates into the nucleus and binds to the small musculoaponeurotic fibrosarcoma (sMaf) protein to form active heterodimers that transactivate downstream antioxidant response elements (AREs) and induce their transcription to exert antioxidant effects (77). Nrf2 is greatly applied to reduce intracellular oxidative stress and apoptosis. Compared to wild-type MDSCs, Nrf2-deficient MDSCs display a greater accumulation of intracellular ROS and attenuated antioxidant enzyme induction (78). MDSCs in the host expressing Nrf2 reduce oxidative stress and cell apoptosis; thus, MDSCs can survive longer (79, 80).

With the exception of Nrf2, the existence of HMGB1 in MDSCs cannot be underestimated. HMGB1, a damage-associated molecular pattern (DAMP) molecule, is a vital driver of MDSC accumulation and immunosuppressive function, as reported in early studies. In the tumor microenvironment, elevated ROS can increase cytoplasmic translocation and release HMGB1 (81). Subsequently, HMGB1 promotes the survival and viability of MDSCs by inducing autophagy (80, 82).

MDSCs also express some enzymes, such as IDO1 and CaMKK2, to negatively modulate the generation of ROS. IDO1, a heme-binding metabolic enzyme, consumes superoxide anion radicals and peroxides to catalyze tryptophan (Trp) into kynurenine (Kyn) (83). CD11b^+^Gr1^+^ MDSCs from IDO-KO hosts enhanced ROS generation and downregulated the expression of ROS scavenging genes (84, 85). Moreover, CaMKK2 could upregulate the transcription level of Nrf2, not NOX1 and NOX2, to decrease the ROS level by phosphorylating and activating its downstream target AMPK (86, 87).

In addition, hypoxia plays a crucial role in regulating the function of tumor derived MDSCs. HIF-1α could decrease NOX2 expression and excessive ROS production, which may give rise to the preferable survival of MDSCs in the tumor microenvironment (88). In turn, ROS could facilitate HIF-1α accumulation, and then HIF-1α activates PDK1, which could prevent the persistence of potentially harmful and superfluous mitochondrial ROS by inhibiting pyruvate dehydrogenase to restrain the conversion of pyruvate to acetyl-CoA, resulting in a lessened tricarboxylic acid (TCA) cycle (89, 90). Apart from hypoxia, tumor cells can increase the glycolysis of MDSCs in the tumor microenvironment. Tumor derived MDSCs displayed higher glycolysis to prevent the cell apoptosis by restraining excess ROS production. Most importantly, the glycolytic metabolite phosphoenolpyruvate (PEP) is a crucial antioxidant agent that averts MDSC apoptosis and contributes to MDSC survival by hindering excessive ROS production (91).

In contrast to MDSCs, HSCs and CSCs have low ROS content. Several signaling molecules, such as ataxia telangiectasia mutated (TAM), PI3K/Akt, FoxO3 (FoxO transcription Factors 3), phosphatase and tensin homology (PTEN), p53, Prdm16 (PR domain-containing 16), HIF-1α, p38MAPK, and Nrf2, account for the low ROS level to maintain stemness and quiescence in HSCs (33). For example, neural stem cells have a high level of ROS (92). CSC cells also have reduced levels of ROS, which may be attributed to the variant isoform CD44v of the adhesion molecules CD44 and CD13 that boosts the activity of the free radical scavenging system (93, 94).

MDSCs can produce ROS by mitochondria and NOX2. MDSCs can take up lipids through FATP2 to promote mitochondrial ROS production. Moreover, the transcription factor STAT3 can increase NOX2 expression to upregulate ROS levels in MDSCs. Then, the elevated ROS level can activate the antioxidant system to eliminate excessive ROS. Nrf2 could be transcriptionally activated to initiate the expression of its downstream antioxidant genes. The high level of ROS can also induce nuclear heterotopic HMGB1 to promote the survival of MDSCs by autophagy. In addition, HIF-1α can activate PDK1 to inhibit mitochondrial ROS production. IDO1 can scavenge ROS with its metabolic characteristics. Another enzyme, CaMKK2, can activate AMPK to decrease ROS production. The glycolytic metabolite PEP could also prevent massive ROS production to keep the ROS level in a suitable range.

## 7 Effects of MDSC-derived ROS

ROS signaling can activate cellular signaling pathways, such as NF-κB, mitogen-activated protein kinase (MAPK), JAK/STAT and phosphoinositide 3-kinase (PI3K)/AKT (4, 95). Furthermore, ROS also enhance the activity of activator protein-1 (AP-1) by stimulating MAPK cascades to dominate a wide range of cellular processes and trigger P53 transactivation that mediates apoptosis, and ROS can induce the expression of redox factor-1 (Ref-1), leading to the transcriptional activity of HIF-1α (96, 97). Generally, ROS are considered to have proinflammatory effects, but it has also been reported that ROS derived from NOX2 have anti-inflammatory functions (45). In the murine arthritis (CIA) model, NADPH-deficient dendritic cells can produce more proinflammatory cytokines and induce both Th1 and Th17 responses to promote autoimmune arthritis (98). In addition, ROS derived from NOX2 could inhibit the NLRP3 inflammasome *via* the PI3K/Akt/NF-κB pathway at 3 days after stroke (99).

**Figure 2:**
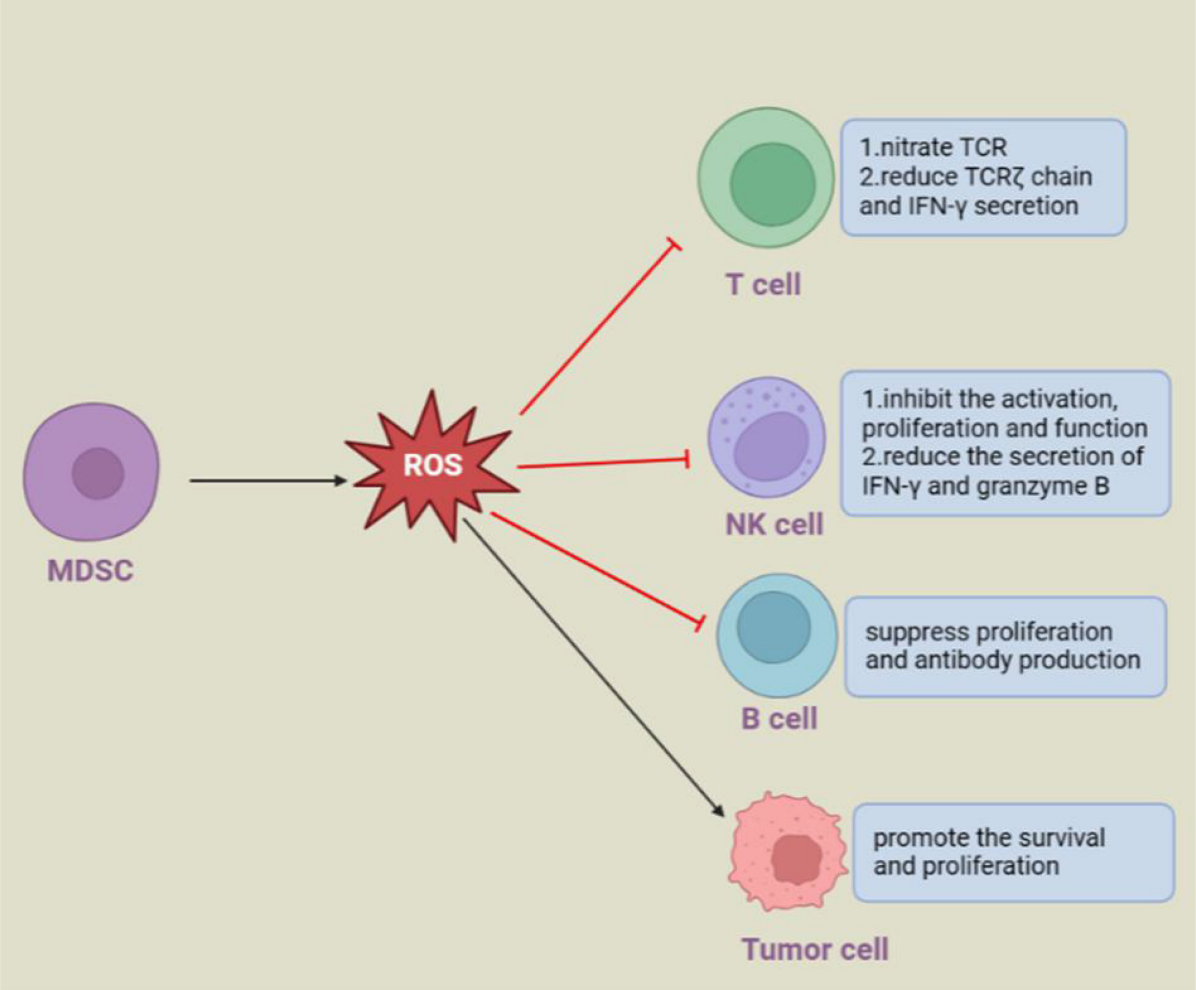
Regulation of other cells by MDSC-derived ROS.

ROS produced by MDSCs could have distinct impacts on different cells (**Figure 2**). In the tumor microenvironment, PMN-MDSCs release ROS into the extracellular space to directly and indirectly support tumor progression. ROS produced by PMN-MDSCs inhibited T-cell responses through p-STAT3 signaling. ROS have an impact on the activation, proliferation and effect of T cells by regulating cell surface thiol levels (44, 100). Specifically, peroxynitrite could nitrate the TCR/CD8 complex, which prevented it from combining with pMHC, and H_2_O_2_ reduced the TCRζ chain and IFN-γ secretion of T cells to destroy T-cell function (43, 48). In addition, when encountering circulating tumor cells (CTCs), PMN-MDSCs can produce excessive levels of ROS to upregulate Notch1 expression in CTCs *via* the Nrf-2-ARE axis. Notch1 could bind to the ligand jagged on the surface of PMN-MDSCs. In addition, Nodal, the downstream target gene of Notch1 in CTCs, can bind to Noda1 recptor cripto in PMN-MDSCs in turn, and the interaction between these signals eventually promotes the survival and proliferation of CTCs (101). Likewise, ROS derived from macrophages and granulocytes can inhibit the activation, proliferation and effect of T cells, and macrophages and activated T cells produce ROS to induce regulatory T cells (102–105).

In addition to inhibiting T cells, ROS released by MDSCs have immunosuppressive activities on B cells and NK cells under infection pathological conditions. During virus infection, two subsets of MDSCs rapidly accumulate at the infected site. In detail, PMN-MDSCs inhibit the activation, proliferation and function of NK cells and reduce the secretion of IFN-γ and granzyme B *via* ROS (106, 107), while M-MDSCs release ROS, including superoxide, peroxynitrite, and nitric oxide, but not H_2_O_2_, to suppress B-cell responses (108). Similarly, human PMN-MDSCs isolated from buffy coats could also produce ROS and other soluble mediators to suppress B-cell proliferation and antibody production (109).

However, professional phagocytes, tumor cells and CSCs are distinct from MDSCs. Professional phagocytes generate ROS to effectively jeopardize pathogens by interacting with microbial components to impair bacterial metabolism (110). ROS in tumor cells have dualistic impacts on the initiation, promotion, progression and metastasis of tumor cells (111). Increased ROS levels in tumor cells could facilitate tumorigenicity by enhancing the proliferation, growth, survival, invasion and metastasis of tumor cells. In contrast to these effects, ROS can suppress tumor growth by inducing apoptosis, autophagy, necrosis and ferroptosis. Both normal stem cells and CSCs exhibit low levels of intracellular ROS content to maintain stemness (112).

In summary, MDSCs and ROS are interactive and mutually beneficial. MDSCs can produce ROS to inhibit antigen-specific T cells (32, 47). In turn, ROS could regulate the differentiation and immunosuppressive activity of MDSCs. In the absence of ROS, the function of MDSCs could be lost to suppress adaptive T-cell responses (43). Additionally, ROS can affect the differentiation of myeloid cells by regulating related gene expression. High levels of ROS can prevent MDSCs from differentiating into mature myeloid cells, while low levels of ROS resulting from catalase and a lack of NOX2 activity enable MDSCs to differentiate into TAMs and DCs (113). How to control ROS levels in MDSCs is a priority and needs further investigation.

ROS produced by MDSCs can have diverse effects on different kinds of cells. MDSCs-derived ROS can promote the proliferation and metastasis of circulating tumor cells by Nrf2/Notch1/Nodal signaling. MDSCs-derived ROS have an inhibitory effect on other immune cells, such as T, B and NK cells, and promote disease progression by inhibiting their function.

## 8 Targeting ROS therapy for MDSCs

Currently, a variety of immune therapies to target MDSCs are being exploited to improve the efficacy of cancer immunotherapy. MDSCs mediated immuno- suppressive function could be abrogated when ROS production is inhibited (114). Remarkable achievements have been made in strategies to lessen the ROS production and block the induction of oxidative stress in MDSCs (115) (**Table 1**).

**Table 1:** Effect of ROS-targeted drugs on MDSCs.

Drug	Type	Disease	Mechanism	References
CDDO-Me	Synthetic triterpenoid	Renal cell carcinoma or soft tissue sarcoma patients EL-4 thymoma, MC38 colorectal carcinoma and Lewis lung cancer mouse model	Activate the target gene NQO1 to decrease ROS level	(116)
Nitroaspirin	Nitro derivative	CT26 colon carcinoma mouse model	Decrease ROS level	(18, 117)
Sanguinarine (SNG)	Benzophenone alkaloid	Lewis lung cancer mouse model	Decrease ROS level	(118)
Baicalein	Traditional Chinese medicine	Systemic lupus erythematosus mouse model	Enhance Nrf2 activation to decrease ROS level	(119)
Jianpi Huayu Decoction (JHD)	Traditional Chinese medicine	H_22_ hepatocellular carcinoma mouse model	Decrease ROS level	(120)
1a,25-Dihydroxyvitamin D3 (calcitriol)	Vitamin D	4-nitroquinoline 1-oxide (4-NQO)–induced esophageal cancer mouse model	Decrease the phosphorylation of STAT3 to decrease ROS level	(121)
Endostatin (ES)	Fragment derived from collagen XVIII	Orthotopic renal cell carcinoma mouse model	Decrease ROS level	(122)
Ferumoxytol	Iron supplement	LPS-induced sepsis mouse model	Decrease ROS level	(123)
L-NIL	iNOS inhibitor	B16 melanoma mouse model	Decrease STAT3 activation to decrease ROS level	(124)
Histamine dihydrochloride (HDC)	NOX2 inhibitor	MC38 colorectal carcinoma and 4T1 mammary carcinoma mouse model	Decrease ROS level in NOX2-dependent way	(125)
Celecoxib	COX-2 inhibitor	AB1 mesothelioma mouse model	Decrease ROS level	(126)
SAHA	Histone deacetylase inhibitor	4T1 mammary tumor mouse model	Increase ROS level	(127)
Alisertib	Aurora-A kinase inhibitor	4T1 mammary tumor mouse model	Downregulate the mRNA expression level of CYBB and NCF1 and inhibit JAK2-STAT3 pathway to decrease ROS level	(128)
Embelin	X-linked inhibitor of apoptosis protein (XIAP) inhibitor	Colitis-associated cancer mouse model	Limit C/EBPβ and STAT3 signaling to decrease ROS level	(129)
Sildenafil	Phosphodiesterase-5 inhibitor	Immunocompetent murine tumor models of major surgery	Decrease ROS level	(130)
N-acetylcysteine (NAC)	ROS inhibitor	P493 B lymphocytoma xenograft mouse model	Stimulate the degradation of HIF-1α to decrease ROS level	(131)
Pam3CSK4	TLR2 agonist	Hepatocellular carcinoma mouse model	Decrease ROS level	(132)
Swertianolin	Isolated from plant gentianella acuta	Sepsis mouse model	Decrease ROS level	(133)
Curcumin	Derived from plant turmeric	Lewis lung cancer mouse model	Decrease ROS level	(134)
Withaferin A (WA)	Natural product	4T1 mammary tumor mouse model	Decrease the phosphorylation of STAT3 to decrease ROS level	(135)
polysaccharide nCKAP-2	Isolated from plant *Curcuma kwangsiensis*	MSC2 cells	Activate TLR4-NF-κB signaling to decrease ROS level	(136)
liposomal doxorubicin and liposomal vaccine containing E75	Liposomal antibiotics and the liposomal peptide	TUBO breast cancer mouse model	Decrease ROS level	(137)
GMI	An immunomodulatory peptide from *Ganoderma microsporum*	*S.aureus*-induced periprosthetic joint infection mouse model	Decrease ROS level	(138)
ApoA-I mimetic peptide 4F (L-4F)	An apolipoprotein A-I (ApoA-I) mimetic peptide	Pancreatic cancer mouse model	Decrease the phosphorylation of STAT3 to decrease ROS level	(139)

The most representative molecules of anti-inflammatory and antitumor drugs are bardoxolone methyl (CDDO-Me), nitroaspirin and Embelin. On account of its capacity to upregulate several antioxidant genes, including NAD(P)H: quinone oxidoreductase 1 (NQO1), thioredoxin, catalase, superoxide dismutase and heme oxygenase, CDDO-Me could efficiently abrogate the immune suppressive effect of MDSCs and enhance T-cell function by activating the target gene NQO1 to decrease MDSC-mediated ROS production, while CDDO-Me did not affect the NO level in MDSCs (116). Nitroaspirin has also been proven to inhibit ROS production and limit the activity of Arg-1 and iNOS in MDSCs (18). Treatment combining vaccination against gp70 with nitroaspirin could inhibit MDSC function and enhance antitumor activity (117). Embelin has anti-inflammatory and antitumor effects in previous studies. It could impair the immunosuppressive activity of MDSCs by reducing the generation of ROS through STAT3 signaling to improve the antitumor immune response in colitis-associated cancer mice (129).

In addition, many inhibitors are being exploited to reduce ROS level such as N-acetylcysteine (NAC), L-NIL, histamine dihydrochloride (HDC), celecoxib, alisertib, SAHA and sildenafil. NAC, a well-established antioxidant that had the ability to reduce ROS and increase the extracellular pool of cysteine. Many animal models have verified its antitumor efficacy. NAC could stimulate the degradation of HIF-1 and inhibit its activity by neutralizing ROS (131). Moreover, the iNOS inhibitor L-NIL, NOX2 inhibitor HDC and COX-2 inhibitor celecoxib could weaken MDSCs function by downregulating ROS production, resulting in enhanced antigen-specific cytotoxicity of CTL (124–126).

Along with inhibitors that target the effector molecules of the immunosuppressive activity of MDSCs, enzyme inhibitors can achieve similar outcomes. The selective Aurora-A kinase inhibitor alisertib directly weakened the immunosuppressive function of MDSCs by notably downregulating the mRNA expression levels of associated genes, such as NOS2, S100A8, S100A9, CYBB and NCF1, and compromising ROS production by inhibiting the JAK2-STAT3 pathway (128). Phosphodiesterase-5 (PDE-5) inhibitors reversed surgery-induced PMN-MDSC immunosuppression by downregulating the level of ROS (130).

Out of the ordinary, the histone deacetylase inhibitor SAHA could augment the intracellular ROS to induce apoptosis in MDSCs. That might be a promising and novel MDSCs-targeted therapy (127). In addition, TLR2 agonist Pam3CSK4 could attenuate hepatocellular carcinoma progression by decreasing ROS content and promoting MDSCs polarization (132).

Natural products are increasingly being discovered and researched in tumor therapy. With the deeper comprehension of natural products, many plant extracts have antioxidant impacts. Among them, withaferin A (WA), a component of the root extract of the plant Withania somnifera Dunal (WRE), could decrease ROS production in MDSCs through a STAT3-dependent mechanism (135). The polysaccharide nCKAP-2 contained in native Curcumae Rhizoma (CR) could induce MDSC apoptosis in a dose-dependent manner through the TLR/NF-κB pathway. In addition, nCKAP-2 can significantly relieve the inhibitory effect of MDSCs on T cells by reducing the ROS level (136). Moreover, curcumin has been reported to lessen the production of ROS and the Arg-1 expression level in MDSCs, which not only inhibited the accumulation of MDSCs in spleen and tumor tissue but also weakened the immunosuppressive function of MDSCs (134). Swertianolin isolated from Swertia and sanguinarine (SNG) derived from Sanguinaria canadensis could prominently decrease the secretion of ROS to inhibit the immunosuppressive effect of MDSCs (118, 133).

Traditional Chinese medicines have made enormous achievements in antioxidant activity. Baicalein is a traditional Chinese herbal medicine. Baicalein prevented the expansion and function of MDSCs in lupus mice, which can be attributed to decreased ROS levels and enhanced Nrf2 activation (119). Jianpi Huayu decoction (JHD), another traditional Chinese medicine, is an experienced prescription for tumor therapy. When MDSCs were treated with JHD, MDSCs could differentiate into macrophages and dendritic cells, and ROS levels were reduced (120).

Furthermore, endostatin (ES) derived from collagen XVIII has the potential to target PMN-MDSCs selectively, resulting in obviously reduced ROS production (122). Doxorubicin (Dox), the conventional chemotherapy to reduce the number of MDSCs in tumor tissues and promote antitumor responses, is converted into a liposomal formulation to improve the efficacy of therapy, as well as the peptide named the E75 epitope (Pep) originating from human epidermal growth factor receptor 2 (HER2/neu). Combination therapy with liposomal nonliposomal Dox and liposomal Pep was the best treatment compared to other single therapies, which decreased ROS generation and downregulated multiple genes related to immunosuppressive function, such as S100A8, S100A9, Arg-1 and iNOS (137). 1a,25-Dihydroxyvitamin D3 (calcitriol) supplementation could reverse the increased level of ROS in IL-6-induced MDSCs (121). In the same way, iron supplementation with ferumoxytol could attenuate MDSC function by significantly downregulating ROS production and inhibiting the expansion of MDSCs in LPS-induced septic mice (123). In addition, GMI is a fungal immunomodulatory protein isolated from *Ganoderma microsporum* that reduces MDSC expansion in bone marrow cells (BMCs) stimulated by *S. aureus* biofilms, which was attributed to increased cytokine expression and a reduction in ROS levels (138, 140, 141). L-4F, an apolipoprotein A-I (ApoA-I) mimetic peptide, inhibited the immunosuppressive function of PMN-MDSCs but not M-MDSCs by decreasing ROS and H_2_O_2_ production (139).

## Conclusion

Based on a previously published review, this paper further updated and listed the new molecules found in recent years that can regulate ROS levels in MDSCs and comprehensively summarized the therapeutic drugs that can target ROS levels in MDSCs. This provides a treatment strategy for cancer immunotherapy.

Compared to other myeloid cells, such as macrophages or tumor cells, ROS play an irreplaceable and distinctive role in MDSCs. On the one hand, MDSCs are required to produce ROS to suppress the antitumor immune response. In turn, excessive ROS can be removed to promote MDSC survival comfortably by activating factors, such as Nrf2, HMGB1, HIF-1α, IDO1, CaMKK2 and PEP. On the other hand, an appropriate level of ROS can prevent further differentiation of MDSCs to better maintain their state and nature. However, other myeloid cells, such as macrophages, have the same sources of ROS as MDSCs and regulate intracellular ROS levels by different factors, such as P2X7 and BAI1. Tumor cells can also induce autophagy to scavenge excessive ROS.

It is widely believed that ROS can promote the development of tumors, but a large number of studies have shown that ROS can promote tumor cell apoptosis and death. At present, studies are emerging that tend to exploit immunotherapies that utilize the ability of ROS to kill tumor cells. Therapy targeting ROS in MDSCs can be therapeutic by impairing MDSC differentiation and function. How to combine targeted ROS therapy in MDSCs and tumor cells requires further consideration.

## Author contributions

JH drafted the manuscript. YZ, KZ and KY discussed and revised the manuscript. SW designed the study and revised the manuscript. All authors contributed to the article and approved the submitted version.
